# Statistical methods to derive efficacy estimates of anti-malarials for uncomplicated *Plasmodium falciparum* malaria: pitfalls and challenges

**DOI:** 10.1186/s12936-017-2074-7

**Published:** 2017-10-26

**Authors:** Prabin Dahal, Julie A. Simpson, Grant Dorsey, Philippe J. Guérin, Ric N. Price, Kasia Stepniewska

**Affiliations:** 1 WorldWide Antimalarial Resistance Network (WWARN), Oxford, UK; 2 Centre for Tropical Medicine and Global Health, Nuffield Department of Clinical Medicine, University of Oxford, Old Road Campus, Roosevelt Drive, Oxford, OX3 7FZ UK; 3 Centre for Epidemiology and Biostatistics, Melbourne School of Population and Global Health, The University of Melbourne, Melbourne, Australia; 4 Department of Medicine, University of California, San Francisco, CA USA; 5 Global and Tropical Health Division, Menzies School of Health Research and Charles Darwin University, Darwin, Australia

**Keywords:** *Plasmodium falciparum*, Kaplan–Meier, Cumulative incidence function, Competing risks, Comparative studies

## Abstract

The Kaplan–Meier (K–M) method is currently the preferred approach to derive an efficacy estimate from anti-malarial trial data. In this approach event times are assumed to be continuous and estimates are generated on the assumption that there is only one cause of failure. In reality, failures are captured at pre-scheduled time points and patients can fail treatment due to a variety of causes other than the primary endpoint, commonly termed competing risk events. Ignoring these underlying assumptions can potentially distort the derived efficacy estimates and result in misleading conclusions. This review details the evolution of statistical methods used to derive anti-malarial efficacy for uncomplicated *Plasmodium falciparum* malaria and assesses the limitations of the current practices. Alternative approaches are explored and their implementation is discussed using example data from a large multi-site study.

**Electronic supplementary material:**

The online version of this article (doi:10.1186/s12936-017-2074-7) contains supplementary material, which is available to authorized users.

## Background

**Fig. 1:**
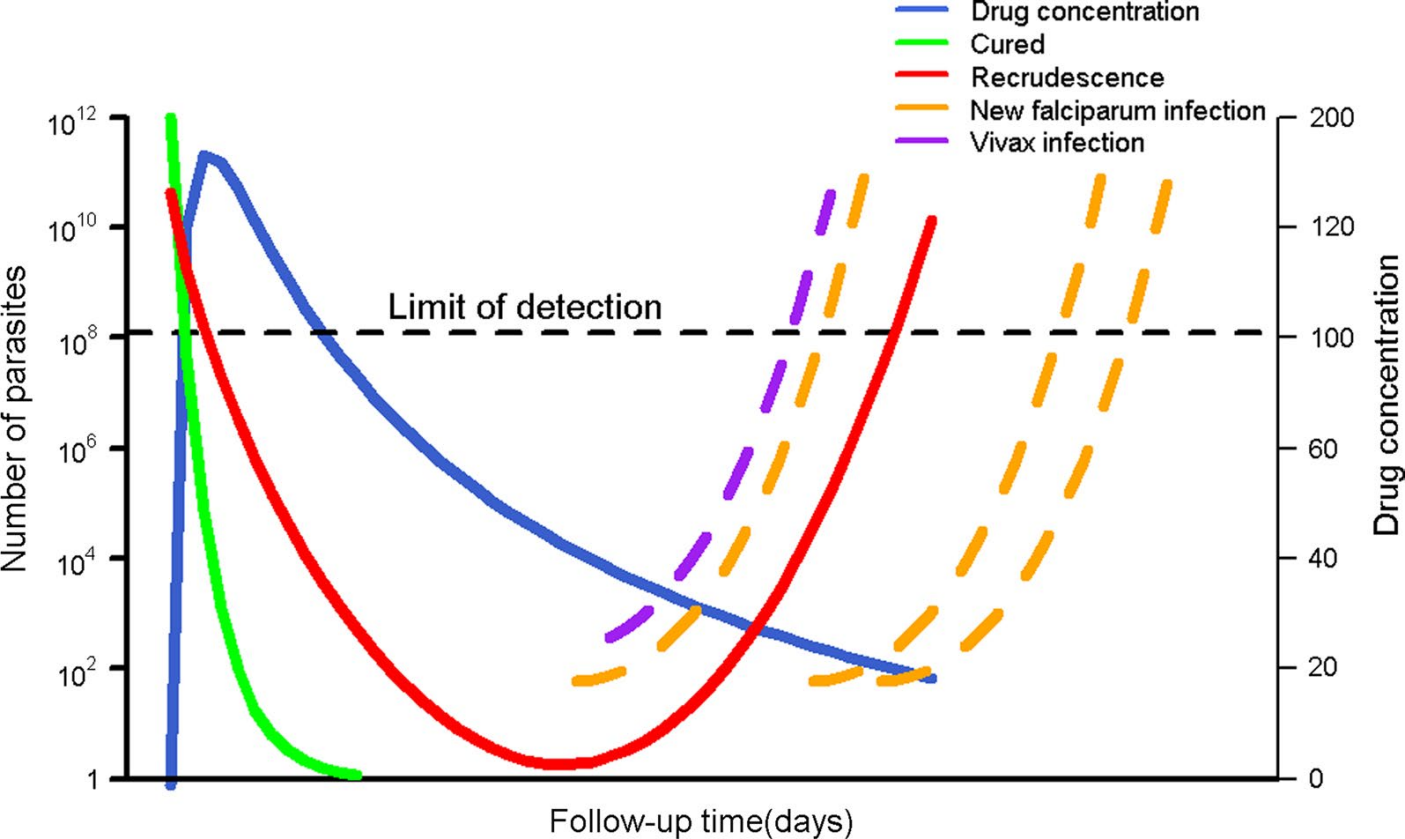
Therapeutic responses post anti-malarial treatment. The blue line represents a hypothetical concentration versus time profile for an antimalarial drug administered orally. The green and red lines represent scenarios for parasite burden versus time profiles following treatment for an infection where all the parasites are completely killed resulting in cure (green) and an infection where parasites are initially killed by high drug levels but with drug levels below the minimum inhibitory concentration (MIC), net parasite growth results in subsequent recrudescence (red). The purple and orange lines represent parasite-time profiles for new infections; either an infection due to a new parasite of the same species (orange) or an infection with a *Plasmodium vivax* parasite (purple) during the follow-up. The left y-axis is for parasite density, and the right y-axis shows drug levels at hypothetical units. The horizontal black line represents the microscopic limit of detection for parasites. The maximum number of parasites a human body can contain is 10^12^ (Adapted from White-2002 [1])

Despite a rich pharmacopeia of anti-malarial agents, the emergence and spread of anti-malarial drug resistance has been relentless, with resistance now documented to all recommended treatment regimens in widespread use. Efficacy estimates derived from in vivo clinical trials, including post marketing and surveillance studies, form the basis for monitoring the status of anti-malarial drug resistance, with in vitro drug susceptibility testing and molecular analyses providing important complementary and confirmatory information. The main measure of anti-malarial clinical efficacy is the risk of recrudescent infection, which is defined as recurrent parasitaemia with parasites that were present prior to the initiation of treatment. Recrudescence needs to be differentiated from reinfections arising from inoculation with a new parasite strain during the follow-up period. New infections can be either from the same parasite species or a different one (Fig. 1) [1].

**Fig. 2:**
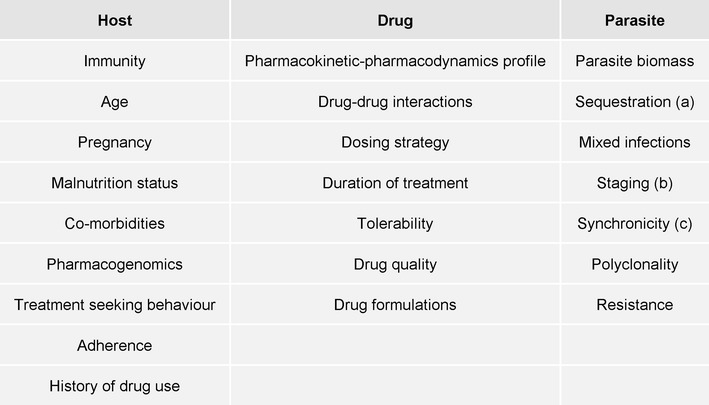
Determinants of in vivo response to anti-malarial treatment. ^a^ The process by which infected erythrocytes containing mature parasites adhere to the microvasculature. Their removal of from the circulation results in the peripheral parasite count being an underestimate of the true parasite biomass. ^b^ The developmental stage of the parasite. The artemisinin compounds have the broadest stage specific action against the parasite. ^c^ Simultaneous rupture of hepatic schizonts result in a uniform stage distribution of the parasite

Recrudescent parasitaemia arises from a variety of factors which can be broadly categorized into those related to the host, the parasite or the drug (Fig. 2). Pharmacokinetic factors can be ameliorated by improved prescribing practices, such as revising the dosing strategy and co-administration with food, whereas significant drug resistance usually requires revision to an alternative treatment regimen. At patient level, failure to achieve adequate clinical cure (i.e. preventing recrudescent infections) can have serious clinical implications including cumulative risk of anaemia, rising parasitaemia, and early treatment failure, which individually and collectively may lead to severe malaria and even death [1]. At the community level, treatment failures can lead to an increased economic burden and onwards transmission, fuelling the development of anti-malarial drug resistance [2].

Anti-malarial efficacy needs to be monitored routinely in endemic areas, so that early indications of drug resistance are recognized and malaria control activities can be revised accordingly [3]. Defining clinical efficacy of current treatment options is a key process in optimizing anti-malarial treatment policy. The World Health Organization (WHO) recommends that treatment efficacy corrected by polymerase chain reaction (PCR) genotyping (defined as 1 minus the risk of recrudescence at day 28) should be at least 90% for existing anti-malarials, and that novel regimens should achieve greater than 95% efficacy to be considered suitable as a first line treatment [4]. However, the classification of treatment outcomes based on PCR-genotyping is known to be vulnerable to its sensitivity, the resolution limits for detecting the differences in the allelic variants of the parasites, the definition used, the number of markers used, the transmission settings, and the genetic diversity of the markers used for sequencing and allele frequencies in different populations [5–8]. In areas of low transmission, multiplicity of infection (MOI) is low (in contrast to the areas of high transmission) [9] and the probability of the pre- and post-treatment alleles being the same due to chance is very small leading to a low misclassification risk for the PCR genotyping. Failure in collecting either the pre-treatment or recurrent parasite DNA isolate will result in missing outcomes. Sometimes, the PCR technique is unable to discriminate recrudescences from new infections due to unsuccessful amplification of DNA or due to failure to interpret the results leading to indeterminate cases. Uncertainty in genotyping procedures leading to misclassification of outcomes has been well studied in anti-malarial literature and outcome classification is vulnerable to the algorithm used and transmission intensity [8, 10]. In addition, study design, the presence of attrition bias, duration of follow-up and the choice of statistical methods to address these confounding factors can have a profound influence on the derived efficacy estimates [11–14].

In this review, the evolution of the methods for defining anti-malarial drug efficacy since the 1960s and the key statistical approaches currently available are documented. Challenges associated with these statistical methods and how they apply to stand-alone efficacy trials and comparative drug studies are discussed.

## Methods for estimating anti-malarial efficacy for a single treatment

**Fig. 3:**
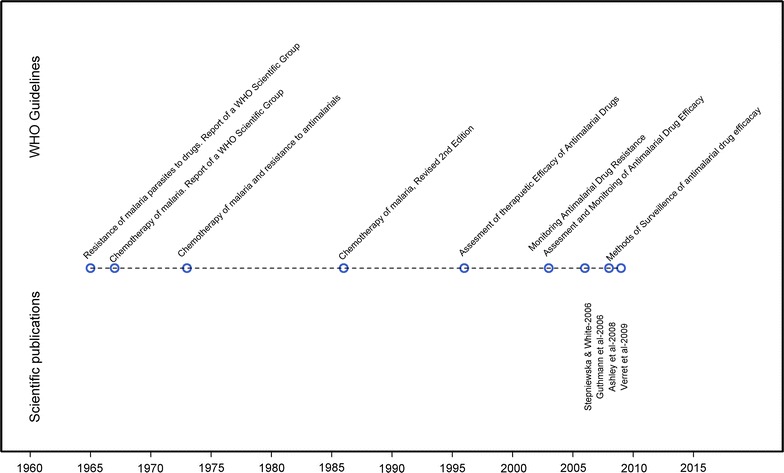
The evolution of guidelines for anti-malarial studies

The WHO released its first standardized protocol for assessing in vivo efficacy against *Plasmodium falciparum* malaria in 1965, primarily to monitor chloroquine resistance which had been identified a few years earlier [15, 16]. This early protocol was revised twice, first in 1967 and then again in 1972, recommending that patients be kept in a mosquito free environment to prevent new infection [17]. These early protocols focused on parasitological outcomes, such as a recurrent peripheral parasitaemia and time to parasite clearance. In the subsequent revisions, methodologies remained largely the same until 1996, when the focus shifted from parasitological response to adequate clinical response [18]. The latter was defined as patients without clinical disease and included those with recurrent parasitaemia but without symptoms. The 1996 WHO protocol focused on parasite clearance assessed on day 14 downgrading the importance of a longer follow-up used for characterizing anti-malarial efficacy—a recommendation retrospectively found to be inadequate [11]. The recommendation were revised again in 2009, when a composite endpoint of both parasitological and clinical assessment was adopted and this was defined as “adequate clinical and parasitological response (ACPR)” [3]. Whilst the earlier guidelines focused on the broader aspects of malarial chemotherapy, the later guidelines have focused more on the methodological aspects of clinical studies. The evolution of these documents and relevant methodical reviews is presented in Fig. 3.

Two approaches have been used in deriving efficacy estimates in anti-malarial efficacy studies: (i) the calculation of the proportion of patients cured within a specified period of follow-up (this proportion is often referred to as the “cure rate”) and (ii) survival analysis, which provides a cumulative probability of cure. The term “cure rate” statistically speaking is misleading since this is not a rate, but a point estimate of risk at a predefined time point. The proportion cured is usually estimated using a per-protocol (PP) and intention to treat (ITT) approaches. In PP approach, the proportion cured is derived from all patients followed until treatment failure or a set period of time, excluding those with protocol deviations, those who develop new infection or who are lost to follow-up. Whilst relatively easy to calculate, this approach ignores valuable information provided by the patients who experience protocol deviations or are lost to follow-up. Patients failing treatment are more likely to become symptomatic and seek retreatment and thus be detected passively. Whereas patients who are cured are more likely to tire from active detection and be lost to follow up—these attrition biases result in an underestimation of treatment efficacy [13]. In a more conservative analysis using an ITT approach, the evaluable population includes all patients enrolled in the study, but patients who are lost to follow-up or who experience protocol deviations are considered as treatment failures. This will underestimate treatment efficacy (and overestimate clinical resistance). This definition of ITT used within the context of anti-malarial studies differs from the standard terminology used in randomized controlled trials, in which ITT generally means that all study participants are included in the analysis as part of the groups to which they were randomized regardless of whether they completed the study or not. Assigning patients who do not complete follow-up the worst possible outcome ensures that derived estimates represent the “worst case scenario”.

Survival analysis using Kaplan–Meier (K–M) method provides an alternative strategy. This approach maximizes the information available from each patient, thereby increasing the precision of the estimates [12, 13]. The K–M estimates of the cumulative probability of cure are usually reported at day 28 and for studies with longer follow-up duration the estimates at the end of the study (e.g. day 42 and 63) are also presented. The complement of K–M (1 minus K–M) is frequently used to derive estimate of the cumulative proportion of treatment failure. In the K–M approach, patients who do not fail treatment during the study period and do not complete follow up for any reasons are included in the analysis until the time of last recorded visit when they are “censored”. Patients who are censored are considered to be at the same risk of experiencing the event of interest as those who continue to be followed, i.e. the censoring is uninformative [19]. Although survival analysis has long been used in other disease areas and in one anti-malarial study in 1995 [20], it was only widely considered for deriving anti-malarial efficacy in 2001 [21] and adopted into the WHO guidelines in 2003 [17]. Stepniewska and White provided a further assessment on the methodological approaches used in anti-malarial studies, and strongly advocated the use of K–M method [12]. A tutorial on deriving efficacy estimates using the K–M survival approach is presented in Additional file 1: Section A.

Several reports have compared the use of PP approach and K–M survival analysis in deriving anti-malarial efficacy. Guthmann et al. pooled datasets from 13 trials (n = 2576) to examine the discrepancies in derived estimates when PP and K–M approach were used [14]. Overall 6% of the samples were lost by day 28 using K–M analysis when indeterminate outcomes were excluded and new infections were treated as treatment success. In contrast, there was a 25% reduction in sample size using PP approach. The risk of recrudescence estimates were lower with the K–M method and the risk differences ranged from − 2.3 to 2.3% when indeterminate cases were excluded. Similar finding was reported by Ashley et al., where the use of PP method was associated with a 34% reduction in sample size as opposed to < 10% reduction when survival analysis was used [22]. In a pooled analysis of 29 clinical trials from Africa and Asia carried out by Verret and colleagues, the PP method consistently overestimated the risk of treatment failure compared to the K–M approach (median difference: 1.7%, range 0–30.9%) and the magnitude of overestimation was proportional to the incomplete follow-up [13]. The authors of these studies recommended the use of K–M analysis, as this minimized the loss of information and made the maximum use of the data.

Since the K–M approach was being increasingly recommended for deriving efficacy estimates, Price et al. provided a classification table for different possible outcomes (see Table 3 of the article) [23]. The WHO guideline published in 2009 further recommended the use of K–M method for deriving anti-malarial efficacy where patients who are lost to follow-up or who develop a new infection during the follow-up period or any other deviations are censored on the day of their last observation in the trial (Table 1) [3]. However, there are several pitfalls and challenges associated with K–M approach that need to be considered [24], and these are addressed in sections to follow, with example from a large multi-centre study carried out in Uganda.

**Table 1:** Assigning outcomes for estimating treatment efficacy under current recommendations. Source: WHO-2009 [3]

End-point for day X (X = 28 or 42)	Cumulative success or failure probability (Kaplan–Meier analysis)	Proportion (per-protocol analysis)
Adequate clinical and parasitological response at day X	Success	Success
Early treatment failure	Failure	Failure
Late clinical failure before day 7	Failure	Failure
Late clinical failure or late parasitological failure on or after day 7		
Falciparum recrudescence	Failure	Failure
Falciparum reinfection	Censored day of reinfection	Excluded from analysis
Other species with falciparum recrudescence	Failure	Failure
Other species infection	Censored day of infection	Excluded from analysis
Undermined or indeterminate PCR	Excluded from PCR-corrected analysis	Excluded from analysis
Loss to follow-up	Censored last day of follow-up according to timetable	Excluded from analysis
Withdrawal and protocol violation	Censored last day of follow-up according to timetable before withdrawal or protocol violation	Excluded from analysis

### Example dataset

Data from a randomized control trial which compared three anti-malarial regimens in four different sites in Uganda from 2002 to 2004 was used as a motivating example [25]. Briefly, 2160 patients aged 6 months or older were randomized to one of the three treatment arms: chloroquine + sulfadoxine–pyrimethamine (CQ + SP), amodiaquine + sulfadoxine–pyrimethamine (AQ + SP) or amodiaquine plus artesunate (AS + AQ). The primary endpoint was the risks of parasitological failure either unadjusted or adjusted by PCR genotyping at the end of the study follow-up on day 28. The study was standardized using the WorldWide Antimalarial Resistance Network (WWARN) clinical protocol [26] and hence the estimates reported in the original article are slightly different to the estimates reported here.

### Challenges in estimating efficacy for a single treatment

#### The presence of competing endpoints

In an anti-malarial trial of uncomplicated *P. falciparum* malaria, the primary endpoint is the risk of recurrence due to reappearance of the same parasite which caused the initial infection (recrudescence). However, patients can experience new infections with *P. falciparum* or other species such as *Plasmodium vivax* during the ensuing weeks (Fig. 1). Such alternative outcomes which can preclude the occurrence of recrudescence are referred to as competing risk events [24]. When studies are conducted in a malaria endemic setting these competing risk events can sometimes occur in over 30% of patients [13]. Once a patient experiences competing events before the end of the study follow-up, recrudescence can no longer be observed as the first event. The presence of competing events changes the number of people remaining at risk of recrudescence and consequently the probability of true treatment failure. In such situations, the overall probability of failing due to recrudescence should be estimated by accounting for the treatment failures due to recrudescence and also recurrence due to the competing events [24].

The K–M method makes a fundamental assumption of independent (non-informative) censoring, i.e. patients who are censored have the same risk of observing the outcome as those who are still being followed-up. When a patient experiences new infections, censoring is no longer non-informative (as they will be retreated) and in such situations the use of K–M leads to an upwards biased estimate of treatment failure [24, 27, 28]. Despite this limitation, the complement of K–M estimate (i.e. 1 minus K–M) is commonly used to derive the cumulative probability of failure in anti-malarial studies. An alternative approach in the presence of competing risk events is the derivation of the Cumulative incidence function (CIF) as proposed by Kalbfleisch and Prentice [29]. CIF estimates the risk of failing from a specific cause at any time between enrolment (*t*_0_) and the time point of interest $\left( {t_{x} } \right)$ and this takes into account the failures from other causes (see Additional file 1: Section A for a tutorial).

**Fig. 4:**
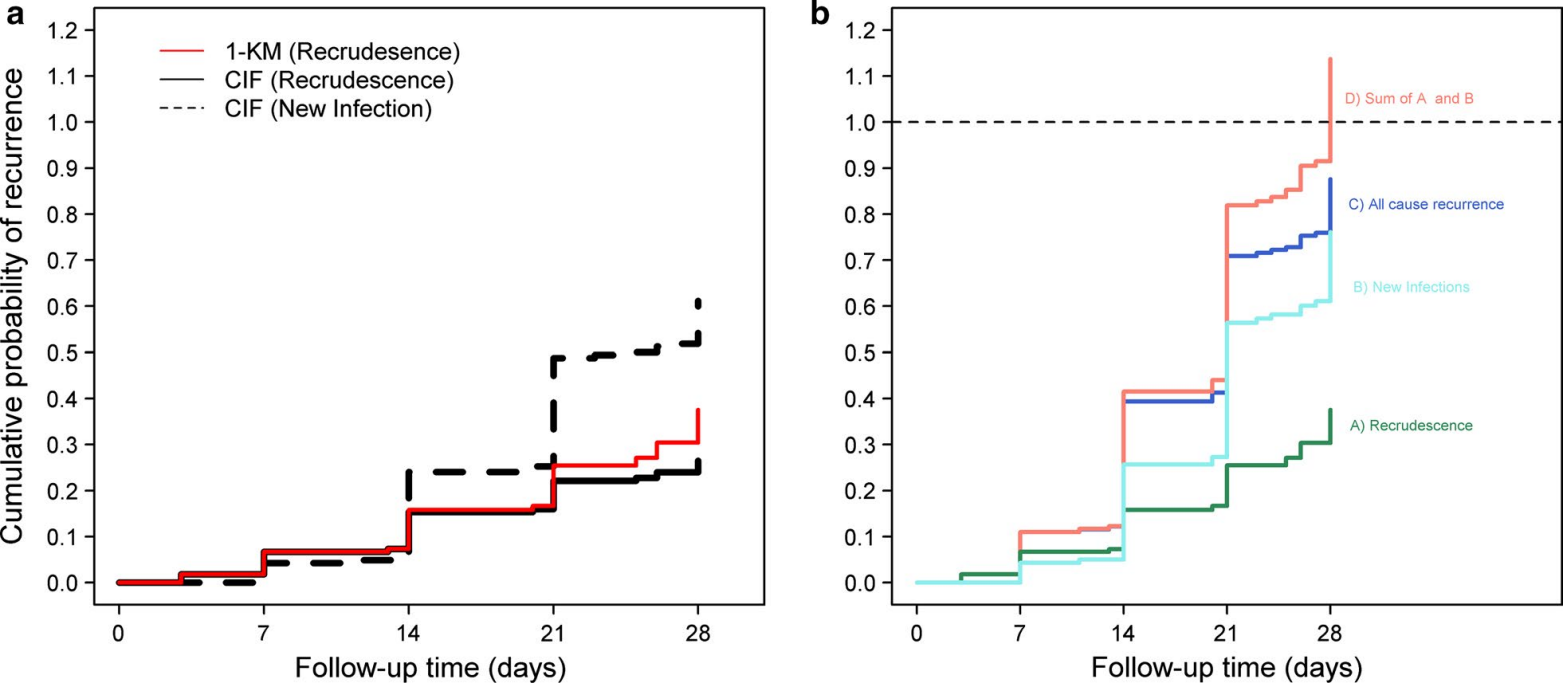
Overestimation of failure using complement of K–M method in Tororo dataset [25]. **a** Cumulative probability of failure due to recrudescence derived using Kaplan–Meier approach (red line) and using Cumulative Incidence Function (solid black line), which accounts for the presence of competing risks (dotted black line). **b** Estimates of the cumulative probability of recurrences for recrudescence (green), new infections (light blue) and overall recurrences (dark blue line) using K–M method. The sum of the probabilities for recrudescence and new infection is presented as the pink line and exceeds the value of 1 at 28 days of follow-up

A comparison of the K–M method and CIF is illustrated using example data from Tororo site in Uganda, an area of high transmission where new infections during follow up were frequently observed [25]. Data from 166 patients treated with CQ + SP were originally analysed using the K–M method and were reanalysed using the CIF approach using *cmprsk* package in R software (Additional file 1: Section B) [30]. The estimate of cumulative probability of recrudescence on day 28 was 0.376 [95% CI 0.264–0.470] using the K–M approach (Fig. 4a) [25], compared with 0.265 [0.199–0.331] calculated using the CIF approach. The corresponding probabilities of reinfections by day 28 were 0.761 [0.667–0.828] using K–M and 0.654 [0.584–0.723] using CIF. These values represent an absolute overestimation of 0.11 for both recrudescence and new infections. In relative terms, this represents an overestimation of treatment failure using K–M approach by 41.8% for recrudescence and 24.4% for new infections. Such overestimation is particularly relevant to new treatment regimens as increasingly high efficacy (> 95%) is demanded for a drug to be considered as a first line therapy. This bias is likely to be worse for regimens which offer only short post treatment prophylaxis in high transmission areas, as new infections are more likely to occur, than for regimens which provide longer protection; this difference in the incidence of new infections over the follow-up period has implications for trials comparing different anti-malarials (comparative trials section to follow). Another challenge with the K–M method is that the sum of individual K–M estimates for different events (e.g. recrudescence and new infection in malaria studies) can exceed the K–M estimate for the composite endpoint of overall recurrence. The sum of the cumulative probability of recurrence estimated using K–M approach for recrudescence and new infection on day 28 was 113.7%, whereas the all cause recurrence estimate was only 87.6% (Fig. 4b). In such situations, the interpretation of K–M estimate as a probability is no longer valid as the sum exceeds 100%.

#### Follow-up data are interval-censored

In anti-malarial studies, active surveillance is generally conducted weekly with follow-up usually scheduled daily for the first 3 days and weekly from day 7 until 28, 42 or 63 days. Thus recurrent parasitaemia, particularly in patients who are asymptomatic, is actively detected and commonly occurs on pre-scheduled follow-up time-points. However, the true timing of microscopically patent positive recurrent parasitaemia is often in between the times of observation, and this gives rise to interval-censored failure times in anti-malarial studies. Ignoring such intervals might lead to an under or overestimation of failures at a given time point, especially when it is assumed that failures occur at the beginning or the end of the interval, and the magnitude of the bias tends to be accentuated as the length of the interval gets larger [31]. Despite this, interval censored data are analysed frequently using K–M method in an ad hoc approach of assuming the failures observed at pre-scheduled visits as the true failure time. Interval censored methods are now part of standard statistical packages and there exists substantial literature on survival estimation and regression methods [32–35]; the algorithm proposed by Turnbull being the most commonly used [32].

The K–M estimates derived using the interval censored method for the Uganda data were similar to the K–M estimates generated by ignoring the interval censoring, and the results and R script for analysis is presented in Additional file 1: Section C.

#### Multi-centre trials

Clinical surveillance studies sometimes enrol patients from more than one centre in order to achieve adequate sample size and sample geographically distinct populations, thus increasing the generalizability of the results. Data are often pooled across sites to get an overall estimate of drug efficacy, but this requires careful consideration. Although samples from different sites are assumed to be independent, there may be heterogeneity in censoring patterns, attrition rates, patient demographics, and transmission intensity. The overall cured proportion at a fixed time point can be computed by combining estimates from each of the sites using standard meta-analysis methods for pooling proportions; the approach of DerSimonian and Laird’s being the most common [36]. However, synthesizing survival curves across sites is challenging. The Cochrane Handbook for Systematic Reviews of Interventions comments on the difficulty in presenting a pooled estimate of K–M from different studies as follows [37]:

*“Kaplan–Meier plots for all pooled participants across trials in a meta*-*analysis have previously been presented in medical journals. This practice breaks with the principle of comparing like with like. For this reason, until further discussions have taken place the Statistical Methods Group is unable to recommend inclusion of such plots in Cochrane reviews.”*

The Cochrane statement is regarding the presentation of the survival curve in a meta-analysis; and this is also relevant for multi-centre studies. However, no specific guidance is provided regarding presentation of point estimates of K–M at specified time points and there exists no consensus among researchers on the best approach to synthesize survival estimates across studies/sites, neither for the aggregate meta-analysis nor for individual patient data meta-analysis. It is common to perform the analysis in a one-step approach where raw data from several sites are pooled as if they came from a single site (naïve approach) and present an overall K–M estimate without considering the multi-centric nature of the data, an approach recommended by Srinivasan and Zhou provided the data from several sites (studies) are independent [38]. However, due to heterogeneity across centres, such approach can result in a treatment appearing to be beneficial after pooling data from several sites, in situations where the reverse is in fact true [39]. Recently, Comberscure et al. proposed a method to pool the K–M estimates from several studies at specific time point [40]. Using this approach, K–M and number of patient at risk are derived for each site at a time-point of interest. DerSimonian and Laird’s (D + L) method is then applied after carrying out an arc-sine transformation of the survival to obtain a pooled estimate of the K–M. This is available through *MetaSurv* package on open source R software [41].

The meta-analytic approach proposed by Comberscure et al. has been illustrated with the example dataset on AS + AQ arm [40]. The K–M estimates for each of the study sites were extracted at the all the pre-scheduled visits (days 1, 2, 3, 7, 14, 21 and 28) including the number of people at risk. The pooled K–M estimates estimated using the naïve approach D + L’s approach are presented in Table 2. The R script for performing analysis is presented in Additional file 1: Section D. In this example, the pooled estimates at day 28 were similar between the two approaches; with the confidence intervals being wider for the estimates derived using D + L’s approach. However, this approach needs to be used with caution as the bias in estimates could be high when sample size per site is small or when the events are rare [40]. In such situation, an alternative approach could be to apply the D + L procedure after complementary log log transformation {log( − log (.))} of the survival function. This approach has the desired statistical property of giving correct coverage probability for the estimated 95% confidence interval of the survival estimate based on as few as 25 observations [42].

**Table 2:** Pooled Kaplan–Meier estimates for recrudescence using naïve and metasurv approaches for AS + AQ [25]

Day	Pooled Kaplan–Meier estimates	
	Naïve approach^a^	D + L approach meta-analysis^b^
7	1.000	0.994 [0.987–1.000]
14	0.994 [0.989–1.000]	0.989 [0.979–0.998]
21	0.949 [0.933–0.966]	0.943 [0.916–0.970]
28	0.918 [0.895–0.941]	0.909 [0.873–0.948]

^a^Kaplan–Meier estimates were estimated assuming the data came from one single study. Patients with new infections, indeterminate outcomes and lost to follow-up were censored when deriving the K–M estimates for recrudescence failures

^b^Kaplan–Meier estimates and associated number of patients at risk were extracted on pre-scheduled follow-up days 1, 2, 3, 7, 14, 21, and 28. The estimates were pooled using *MetaSurv* package in R (Additional file 1: Section D). I squared statistic for heterogeneity = 0%

#### Challenges specific to comparative trials

To investigate suitable alternative treatment regimens, a comparative randomized clinical trial is required and these comparative trials raise further difficulties in the analyses and interpretation of the data.

#### Comparison of anti-malarial drugs with different pharmacokinetic profiles

**Fig. 5:**
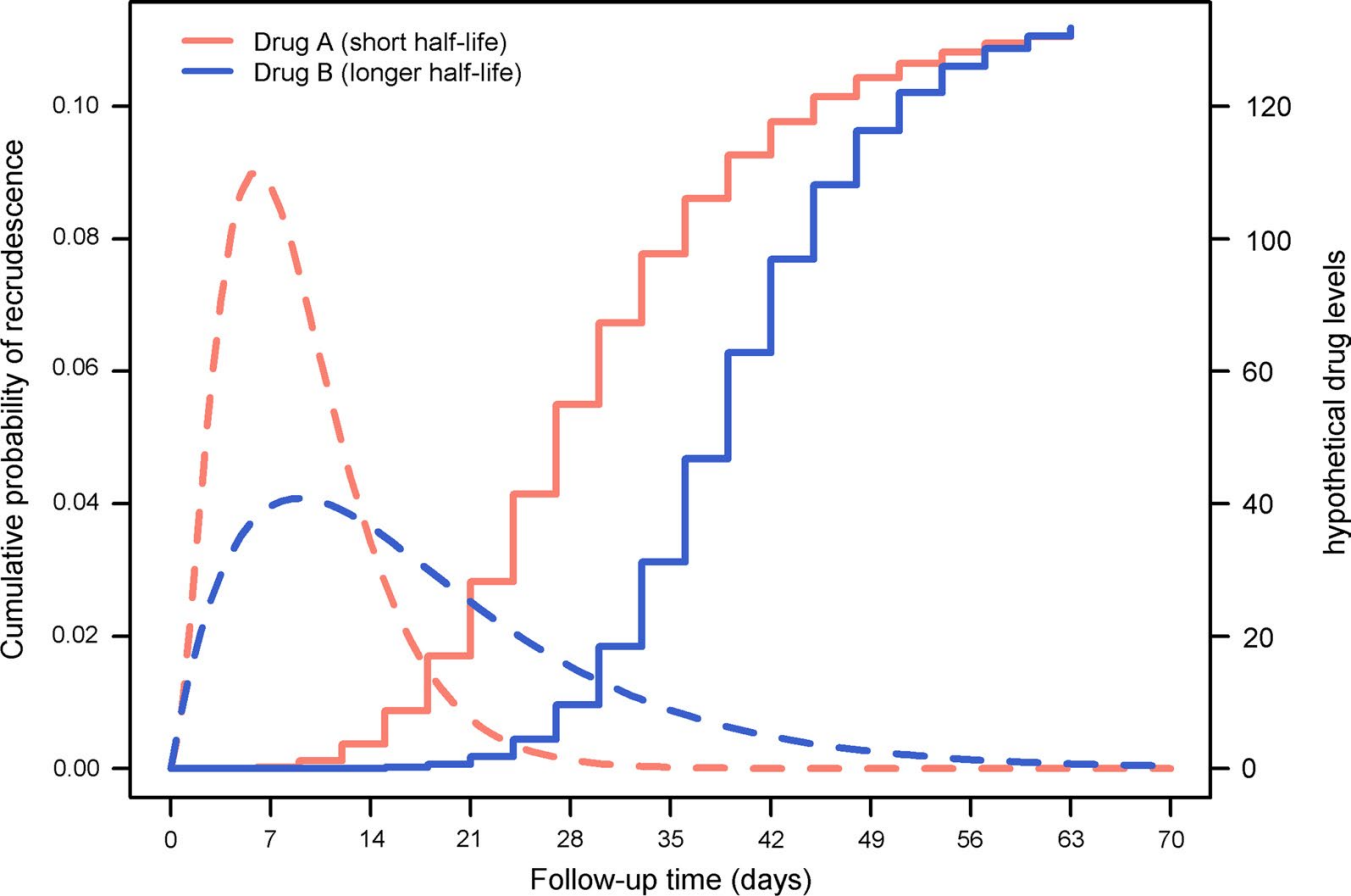
Cumulative failure estimates for drugs with different terminal elimination half-lives. The solid line represents K–M cumulative failure probabilities and the dotted line represents the drug levels with hypothetical units presented on the right y-axis. Drug B (blue) has a longer elimination half-life and the recrudescent failures are more patent after day 21 compared to drug A (red) with a short half-life and recrudescences being observed after day 7

Anti-malarial drugs with different elimination half-lives may vary considerably in the period of time during which peripheral parasite growth is suppressed. Since post treatment prophylaxis reduces the risk of new infection and delays the timing of recurrent parasitaemia, for drugs with similar efficacy the comparative results may be biased against the drug with the shorter half-life. Conversely, a new drug which has a long elimination half-life may result in the false impression of good efficacy. Consider dihydroartemisinin–piperaquine (DP) and artemether–lumefantrine (AL). Piperaquine is a slowly eliminated regimen with a terminal elimination half-life of 13.5–28 days and will suppress parasitic growth for a far longer period compared to lumefantrine, which has an elimination half-life ranging from 1.4 to 11.5 days [4]. Patients treated with DP will therefore encounter fewer recrudescences/new infections during the first 28 days of the follow-up and thus comparison of efficacy at day 28, will be biased against artemether–lumefantrine (AL; see Fig. 5 for an illustration of this bias). For example, in a longitudinal study conducted in Uganda, the median time to recrudescence was 21 days (range 21–50 days) in the AL arm compared to 42 days (range 33–51 days) in the DP arm [43].

Ideally, the comparison overall efficacy of anti-malarial regimens should be carried out at a time, when anti-malarial drug concentrations cease to suppress parasite growth (i.e. fall below the minimum inhibitory concentration—MIC) and after allowing the time for the parasites to reach the limit of detection. If the drug concentrations fall below MIC on day 18, and assuming 10 parasites are circulating in the blood, these parasites will reach the threshold for detection (~ 10^8^ parasites) in 7 parasite life cycles, which is approximately 14 days assuming an efficient multiplication of tenfold per cycle and a parasitic developmental cycle of 48 h. In this scenario recrudescences begin to appear by day 33. Under the same assumption, a drug which provides more prolonged prophylaxis (e.g. with drug concentrations in plasma falling below than MIC on day 31) will result in recrudescent parasites reaching the limit of detection on day 45. Hence, comparing these two drugs on day 42 will result in a biased conclusion. The comparative efficacy should account, therefore, for differences in the pharmacokinetic profile of the drugs. This is further confounded by transmission setting, which determines the risk of new infection (a competing risk event). Characterization of the duration of follow-up required to appropriately capture the treatment failures would provide a basis for comparison and this is currently being investigated [44].

##### Comparing survival estimates at a fixed point in time in a single centre study

Comparison of survival curves is usually carried out using the log-rank test. In competing risk analysis situation, such comparison is made using Gray’s test [45]. The log-rank test uses information throughout the study follow-up period with equal weights given to failures at all time points. This is the most powerful test under the assumption of proportional hazards. Intersection of two survival curves may be indicative of non-proportional hazards and the log-rank test will fail to pick up differences. In such situation Gehan’s test and non-parametric tests such as Kolmogorov–Smirnov and Cramer–von Mises types of tests may be used [42]. Which of these two tests (log-rank or Gray’s test) remains the appropriate approach has gathered considerable interest in statistical literature. It has been suggested that if the interest is in understanding the biological mechanism (e.g. how a treatment affects recrudescence), the log rank test is considered appropriate and when a researcher is interested is answering if subjects receiving a particular drug are more likely to fail (e.g. experience recrudescence by the end of the study), the comparison of CIF through Gray’s test is considered appropriate [24, 46, 47].

In addition, when comparing two anti-malarial regimens (e.g. AL and DP) it appears to be more relevant to focus on the overall proportion of failures observed during the follow-up time. Consequently, an alternative approach test is needed which allows comparison of the cumulative Kaplan–Meier estimates at a specific time point. Such a test can be constructed from the difference of complementary log–log transformed K–M estimates at a specified time point and the appropriately estimating the standard error for this difference [42].

**Fig. 6:**
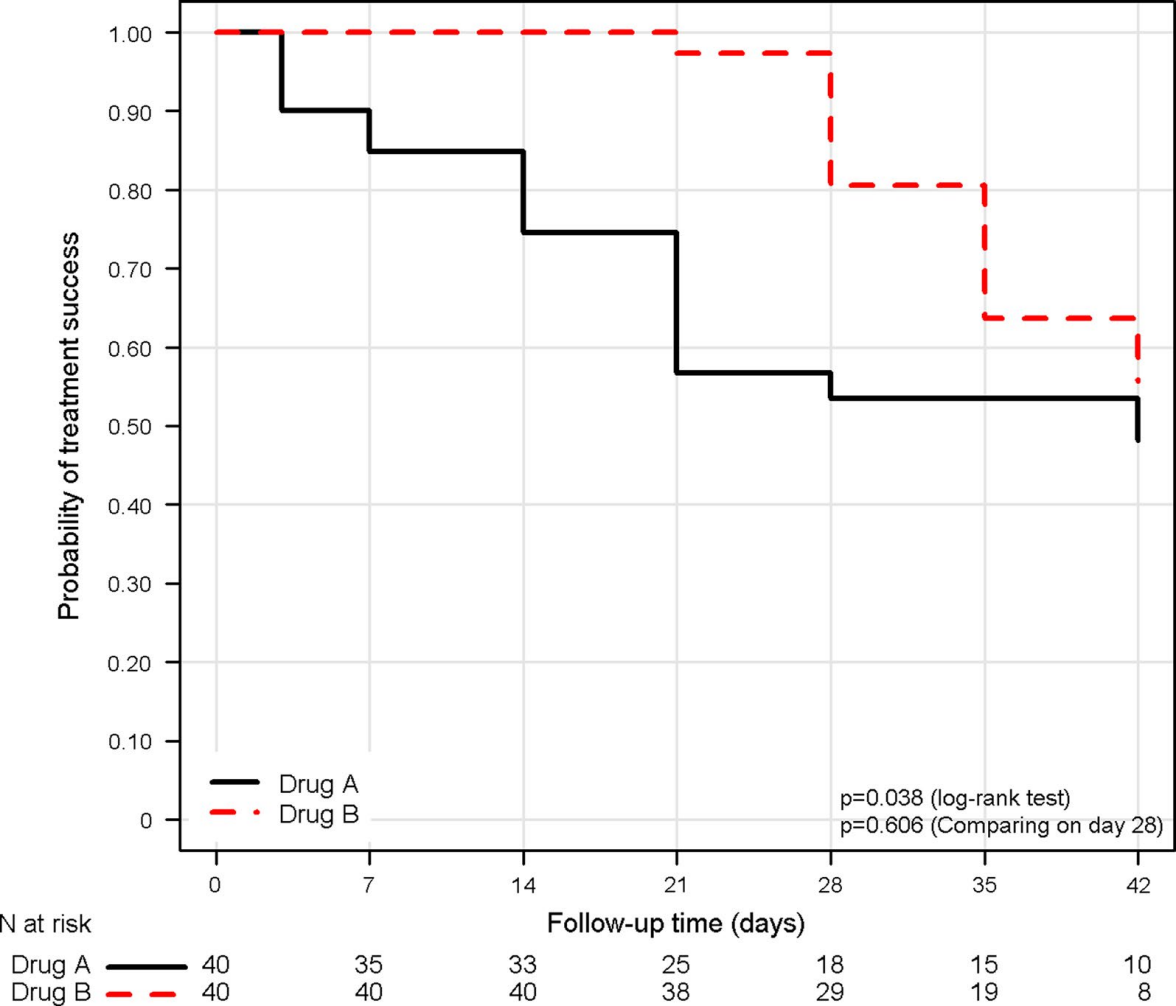
Comparison of survival estimates at fixed point in time

Let $\hat{S}1\left( t \right)$ and $\hat{S}2\left( t \right)$ be the two survival estimates and $\delta = \{ \ln \left( { - \ln \left( {\hat{S}1\left( t \right)} \right)} \right) - \ln \left( { - \ln \left( {\hat{S}2\left( t \right)} \right)} \right)$ be the difference between these two estimates on complementary log–log scale. The *X* ^2^ test statistic for the comparing the difference in two estimates is given by [42]:

$$X^{2} = \frac{{\{ \delta \}^{2} }}{Var\left( \delta \right)}$$

where *Var*(*δ*) denotes variance estimate of *δ* (see Additional file 1: Section E for details of derivation and R associated script). This statistic has an approximate Chi squared distribution in 1 degree of freedom. The approach recommended by Klein et al. [42] (referred as Klein’s test onwards) has been illustrated using example data 2 provided in Additional file 1: Section E. There was a significant difference between drug A and drug B on day 42 based on log-rank test (${\rm X}^{2} = 4.30$, p = 0.038). Using Klein’s test, there were no difference in the K–M estimates on day 42 (X^2^ = 0.266, p = 0.606) (Fig. 6).

##### Comparing survival estimates at a fixed point in time in multicentre trials

Klein’s test to compare survival estimates at a fixed point in time can be extended to multi-centre studies (Additional file 1: Section E) [42]. The result of the Klein’s test and the stratified log-rank test for comparing AQ + SP against AS + AQ were similar and are presented in Table 3.

**Table 3:** Comparing K–M at fixed point in time

Site	Chi squared test for comparing K–M at a fixed time point (day 28) (Klein’s test [42])		Log-rank test for comparing whole curve (day 0 to day 28)	
	*X* ^2^	*p* value	*X* ^2^	*p* value
Tororo	1.35	0.246	2.00	0.158
Arua	2.72	0.099	2.20	0.142
Jinja	6.18	0.013	6.40	0.011
Apac	0.91	0.340	1.10	0.290
Stratified test	4.98	0.026	4.90	0.026

#### Non-inferiority designs

Historically clinical trials have been designed to determine the superiority of a new regimen against that of a failing drug [48]. This approach is useful when the efficacy of the standard regimen has already reached unacceptable levels. However, when comparing highly effective regimens it is often unfeasible to demonstrate the superiority since the required sample size will be extremely large. In this scenario a non-inferiority design is often adopted [12]. The primary objective of a non-inferiority trial is to demonstrate that an investigational drug regimen is not clinically inferior (“is no worse”) to the current standard of care. This can be demonstrated by showing the two-sided 95% confidence interval (CI) for treatment difference is likely to lie above a lower margin of clinically acceptable differences (Δ). Currently, there exists no recommendation regarding the optimal Δ margin for comparative studies. The US Food and Drug Administration (FDA) requires construction of two-sided 95% confidence interval for the difference in cure with a pre-specified Δ for the per-protocol and modified ITT population to demonstrate non-inferiority [49]. For anti-malarial studies, Borrmann et al. recommend using a non-inferiority margin of 5% units (or, its equivalent as hazards ratio unit) for phase III trials provided that the cured proportion remain above 90% [48]. Since anti-malarial efficacy with an ACT regimen is invariably close to or greater than 95%, a non-inferiority margin of 5% can be regarded as a reasonable choice. Smaller margin will require a much larger sample size, which has immediate implication on cost and resources. A larger margin will lead to a smaller sample size but would not guarantee that the efficacy of the comparator is greater than 90%.

Currently the demonstration of non-inferiority is widely based on the cured proportion. Since the K–M method is the recommended statistical approach for deriving anti-malarial efficacy, any demonstration of non-inferiority should be based ideally on differences in the K–M estimates. This can be carried out a fixed point in time or can be based on the whole curve.

##### Demonstrating non-inferiority based on K–M estimates at a fixed point in time

The hypothesis of non-inferiority at a fixed time point (e.g. day 28) can be tested using the K–M estimates. Suppose Δ is the margin for non-inferiority. The 95% CI for the difference of two K–M estimates can be constructed by adding individual variance of the two K–M estimates [50]. For anti-malarial studies, Stepniewska and White have proposed the use of effective sample size for constructing 95% CI for differences in K–M estimates and this can be easily applied for any trial [12]. The effective sample size is calculated by dividing the derived K–M estimates at a given time point by the number of patients who reached the end of the study without any deviations or treatment failure (see Additional file 1: Section F for a worked out example).

##### Demonstrating non-inferiority based on relative risk measure

Under the proportional hazards assumption, the non-inferiority between regimens can be tested based on the relative risk measure (e.g. hazards ratio). Let $\hat{S}1\left( t \right)$ and $\hat{S}2\left( t \right)$ be survival estimates for the new and standard treatment regimens respectively at time *t*. Let *γ* be the corresponding relative risk of failure for new treatment compared to the standard regimen (estimated as hazards ratio from Cox’s model) and let $\Delta = \hat{S}1\left( t \right) - \hat{S}2\left( t \right)$ be the non-inferiority margin. Under the assumption of proportional hazards [51],

$$\hat{S}1\left( t \right) = [\hat{S}2\left( t \right)]^{\gamma } \Rightarrow \gamma = \frac{{\ln \left( {\hat{S}1\left( t \right)} \right)}}{{\ln \left( {\hat{S}2\left( t \right)} \right)}}$$

The upper limit for non-inferiority based on relative risk then can be derived based entirely on the efficacy of the reference (standard) arm as:

$$\gamma = \frac{{\ln \left( {\hat{S}2\left( t \right) - \Delta } \right)}}{{\ln \left( {\hat{S}2\left( t \right)} \right)}}$$

**Fig. 7:**
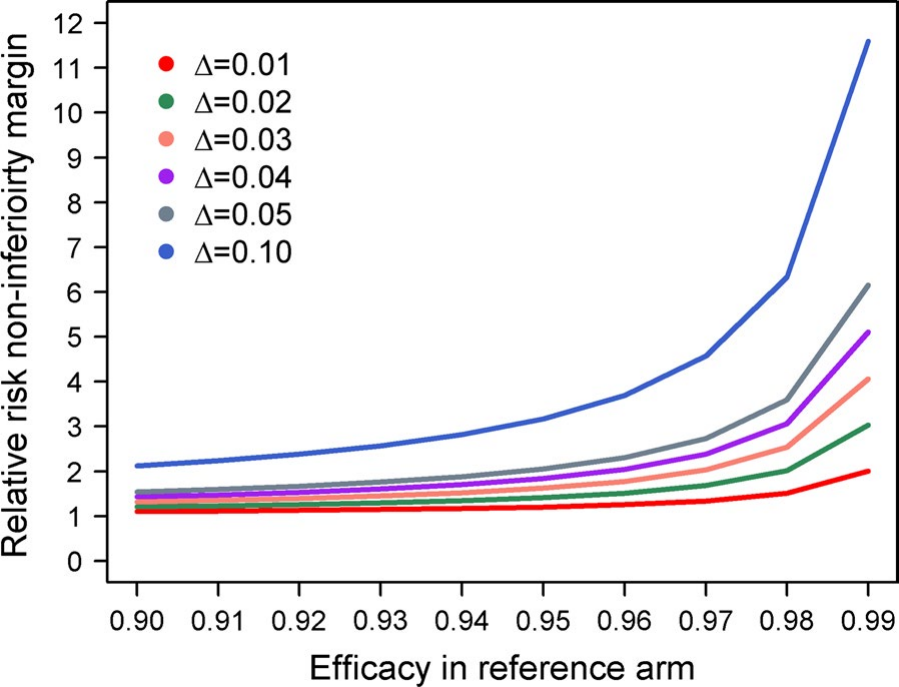
Upper limit of non-inferiority margin based on the relative risk. Relationship between non-inferiority margin on the relative risk scale, and the margin on the survival difference (difference of two Kaplan–Meier estimates) scale, Δ

Non-inferiority is demonstrated if the 95% CI for the estimated hazards ratio remains below the non-inferiority limit on the relative risk scale [48]. Further recommendations on interpretation of the results of the non-inferiority tests for Phase III studies is provided elsewhere (see Table 1 of Bormann et al. [48]). Figure 7 shows the relative risk non inferiority margin corresponding to survival estimates > 90% in the reference treatment (Additional file 1: Section G). If 5% difference is considered an appropriate non-inferiority margin in K–M scale, then on relative risk scale, when the existing treatment has an efficacy of 97%, the upper limit of the 95% CI for the derived hazards ratio for the new treatment should not exceed 2.7. One advantage of demonstrating non-inferiority on the relative risk scale as opposed to absolute differences in K–M is that the influence of baseline covariates can be adjusted when deriving the hazards ratio using Cox’s regression model as outlined by Tunes da Silva et al. [51]. However, one should be careful when performing such comparison between regimens with different half-lives and must validate the assumption of hazards being proportional. When the assumption of proportionality doesn’t hold, an alternative approach is to construct the test based on the complementary log–log transformation of the two survival estimates at time *t*. Logarithm of the hazard ratio corresponds to the difference between K–M survival estimates on the complementary log–log scale. The 95% confidence interval (on the complementary log log scale) for this difference can be calculated using expression in Additional file 1: Section E. The estimated confidence interval can then be transformed into a relative ratio (hazard ratio) scale by exponentiation (a working example is presented in Additional file 1: Section H).

## Conclusions

Significant resources have been spent to overcome recognized difficulties in estimating anti-malarial efficacy by standardizing methodological procedures and these have facilitated the monitoring of anti-malarial drug resistance over time and geographical location. However, even a robust design and analysis requires that the derived point estimates of efficacy are interpreted with caution and the confidence interval around such point estimates should be given equal importance when interpreting the result of a study. The study design, transmission setting, laboratory and genotyping procedures, patient demographics and adherence to protocols all need to be considered. Uncertainty associated with PCR genotyping can be ameliorated to a certain extent if allelic distribution and clonality of infection in the study population is known. This allows for adjustment of pre and post treatment alleles matching purely by chance; various modelling approaches have been proposed for estimating the haplotype frequencies and for adjusting drug efficacy estimates and novel genotyping approach has been recently suggested [10, 52–56]. For comparative studies, the results are confounded by the elimination half-lives of the drugs and the transmission setting in which the study is conducted. Advances in statistical methodologies and the availability of the methods in standard software programs have ensured that some of the issues raised in this review can now be easily addressed (Table 4). However, the true extent of the problem associated with these limitations is likely to have been overlooked, especially when individual trials are small and failures are few. It is important to define the extent of this bias, as derived efficacy estimates form the basis for driving policy decisions. The remit of the WorldWide Antimalarial Resistance Network Methods Study Group is well placed to answer some of these issues that will facilitate better methodologies and practices [44].

**Table 4:** Challenges in estimating antimalarial drug efficacy and possible alternatives

Challenges	Current approach	Alternative approach	Software
Competing risk event	Censored on the day of event [3]	Cumulative incidence function	*cmprsk* package R [30] (Additional file 1: Section B)
Interval censoring	Ignored	Interval censored survival estimates	*survival* package R [57] (Additional file 1: Section C)
K–M for multicentre studies	No specific recommendation	Use of meta-analysis approach [40]	*MetaSurv* package R [41] (Additional file 1: Section D)
Comparing K–M estimates	Current comparison based on whole survival curve (log-rank test)	Comparison at a fixed point in time based on complementary log–log transformation [42]	R script available as additional file (Additional file 1: Section E)
Demonstrating non-inferiority	Based on the cured proportion	Based on the difference of two K–M estimates after complementary log–log transformation (or on hazards ratio scale)	R script available as additional file (Additional file 1: Sections F, G, H)

## Additional file

**Additional file 1.** Additional tutorial, formulae and results.
