# Supplementary material for: Statistical methods to derive efficacy estimates of anti-malarials for uncomplicated Plasmodium falciparum malaria: pitfalls and challenges
Source: Malar J. 2017 Oct 26;16:430. doi: 10.1186/s12936-017-2074-7 (PMC5658934; doi:10.1186/s12936-017-2074-7)
Supplement: Supplementary file 1 — Additional file 1. Additional tutorial, formulae and results. [file 12936_2017_2074_MOESM1_ESM.docx]

**Additional file 1_additional tutorial, formulae and results for**

**Statistical methods to derive efficacy estimates of anti-malarials for uncomplicated *Plasmodium falciparum* malaria: pitfalls and challenges**

Prabin Dahal, Julie A Simpson, Grant Dorsey, Philippe J Guerin, Ric N Price, Kasia Stepniewska

**Section A: Worked out example on estimating antimalarial efficacy**

Suppose the following data were observed in a trial of 20 patients, where 4 patients had recrudescence, 10 patients had new infections and 6 patients reached the administrative end of the study without any parasite recurrence. The derived failure estimate using per protocol approach was 0.40, 0.28 using Kaplan Meier analysis and 0.20 using cumulative incidence function (CIF).

Example data 1

| **Day** | **Outcome** |
| --- | --- |
| 14 | Recrudescence |
| 14 | Recrudescence |
| 15 | New infection |
| 17 | New infection |
| 22 | New infection |
| 22 | New infection |
| 28 | New infection |
| 28 | Recrudescence |
| 33 | New infection |
| 34 | New infection |
| 44 | New infection |
| 52 | New infection |
| 61 | New infection |
| 63 | Recrudescence |
| 63 | Adequate clinical and parasitological response |
| 63 | Adequate clinical and parasitological response |
| 63 | Adequate clinical and parasitological response |
| 63 | Adequate clinical and parasitological response |
| 63 | Adequate clinical and parasitological response |
| 63 | Adequate clinical and parasitological response |

1. **Per protocol proportion**

In a study, the proportion pf patients with treatment failure at time *t* can be computed as:

$$p=\frac{d}{n}$$

where $d$is the total number of recrudescence failures, and $n$ is the total number of patients who reach the end of the study who weren’t loss to follow-up or didn’t deviate from the protocol.

Under per protocol approach, patients who experience new infections are excluded from the analysis. The overall failure estimate would then be total number of recrudescence divided by the number of the patients who reach end of the study with or without recrudescence failures. For this example, the failure proportion is 40% (4/10). Conversely, the cured proportion is 60%.

1. **Kaplan-Meier (K-M) approach**

Estimate of cumulative proportion of patients with cure is computed as

$$K‒M\left( t \right)=\prod_{k\leq t} \frac{n_{k}-d_{k}}{n_{k}}$$

where $d_{k}$is the number of recrudescences at time *k* and $n_{k}$ is the total number of patients at risk of experiencing recrudescence just before time *k*, and the symbol $\Pi$ presents the product over all distinct times when recrudescences or losses to follow-up were observed. The failure estimate is given by 1 minus the K-M. The value of K-M (t) remains constant between the times of recurrent infections; thereby the estimated survival probability is a step function that only changes value at each event time. The number at risk of recurrence is all patients at time zero, and then is reset at each event time t to exclude any patients who had parasite recurrence or were loss to follow-up before time t. The K-M estimates are presented in Table 1.

1. **Cumulative incidence function (CIF)**

Cumulative incidence function at time *t* for event *j* is defined as

$${CIF}_{i}(t)=\sum_{k\leq t} \frac{d_{ik}}{n_{k}}.\hat{S}(k-1)$$

Where $d_{ik}$ is the number of events of type $i$ that occur at time $k$, and $n_{k}$ is the number at risk at time $k$, and $\hat{S}(k-1)$ is the K-M estimator of the survival probability of being free of any event type prior to time $k$. This shows that CIF for any event type not only depends on the number of individuals who have experienced the event of interest, but also on the number of individuals who haven’t experienced any other type of events. Thus, calculation of CIF accurately acknowledges the presence of competing risk events. Calculation of the CIF is presented in Table 2.

**Table 1: Kaplan-Meier approach for estimating recrudescence failure**

| Time – t (days) | Number at risk of recrudescence  just before time t – n_t_ | Number with recrudescence at time t - d_t_ | Number censored at time t | K-M Survival probability at time t  S(t)=S(t-1)(1-d_t_/n_t_) | 1-KM |
| --- | --- | --- | --- | --- | --- |
| 0 | 20 | 0 | 0 | 1.000 | 0.000 |
| 14 | 20 | 2 | 0 | 1 x (1-2/20)=0.900 | 0.100 |
| 15 | 18 | 0 | 1 | 0.9x (1-0/18)= 0.900 | 0.100 |
| 17 | 17 | 0 | 1 | 0.9x (1-0/17)=0.900 | 0.100 |
| 22 | 16 | 0 | 2 | 0.9x (1-0/16)=0.900 | 0.100 |
| 28 | 14 | 1 | 1 | 0.9x (1-1/14)=0.836 | 0.164 |
| 33 | 12 | 0 | 1 | 0.9x (1-0/12)=0.836 | 0.164 |
| 34 | 11 | 0 | 1 | 0.9x (1-0/11)=0.836 | 0.164 |
| 44 | 10 | 0 | 1 | 0.9x (1-0/10)=0.836 | 0.164 |
| 52 | 9 | 0 | 1 | 0.9x (1-0/9)=0.836 | 0.164 |
| 61 | 8 | 0 | 1 | 0.9x (1-0/8)=0.836 | 0.164 |
| 63 | 7 | 1 | 6 | 0.9x (1-1/7)=0.716 | 0.284 |

**Table 2: Cumulative incidence function (CIF) for estimating recrudescence failure in the presence of competing risk due to new infections.**

| Time  days | N at risk  from any event | Type of event | Overall survival probability  using K-M (any recurrence included) | Proportion of recrudescence | CIF (recrudescence) | Proportion of  new infection | CIF (new infection) |
| --- | --- | --- | --- | --- | --- | --- | --- |
| 0 | 20 | - | 1.000 | 0/20 = 0.00 | 0.00 | 0/20 = 0.00 | 0.00 |
| 14 | 20 | 1 | 0.900 | 2/20 = 0.10 | 0.00+ (0.10*1.000) = 0.10 | 0/20 = 0.00 | 0.00 + (0.00*1.000) = 0.00 |
|  | 20 | 1 |  |  |  |  |  |
| 15 | 18 | 2 | 0.850 | 0/18 = 0.00 | 0.10+ (0.00*0.900) = 0.10 | 1/18 = 0.05 | 0.00+ (0.10*0.900) = 0.05 |
| 17 | 17 | 2 | 0.800 | 0/17 = 0.00 | 0.10+(0.00*0.850) = 0.10 | 1/17 = 0.06 | 0.05+(0.06*0.850) = 0.10 |
| 22 | 16 | 2 | 0.700 | 0/16 = 0.00 | 0.10+ (0.00*0.800)= 0.10 | 2/16 = 0.13 | 0.10+(0.13*0.800) = 0.20 |
|  | 16 | 2 |  |  |  |  |  |
| 28 | 14 | 2 | 0.600 | 1/14 = 0.07 | 0.10+(0.07*0.700)= 0.15 | 1/14 = 0.07 | 0.20+(0.07*0.700) = 0.25 |
|  | 14 | 1 |  |  |  |  |  |
| 33 | 12 | 2 | 0.500 | 0/12 = 0.00 | 0.15+(0.00*0.600) = 0.15 | 1/12 = 0.08 | 0.25+ (0.08*0.600) = 0.30 |
| 34 | 11 | 2 | 0.500 | 0/11 = 0.00 | 0.15+(0.00*0.500) = 0.15 | 1/11 = 0.09 | 0.30+ (0.09*0.500) = 0.35 |
| 44 | 10 | 2 | 0.450 | 0/10 = 0.00 | 0.15+(0.00*0. 500) = 0.15 | 1/10 = 0.10 | 0.35 +(0.10*0.500) = 0.40 |
| 52 | 9 | 2 | 0.400 | 0/9 = 0.00 | 0.15+(0.00*0.450) = 0.15 | 1/9 = 0.11 | 0.40+ (0.11*0.450) = 0.45 |
| 61 | 8 | 2 | 0.350 | 0/8 = 0.00 | 0.15+(0.00*0.400) = 0.15 | 1/8 = 0.13 | 0.45+ (0.13*0.400) = 0.50 |
| 63 | 7  7  7  7  7  7  7 | 1 | 0.300 | 1/7 = 0.14 | 0.15+(0.14*0.350)= 0.20 | 0/7 = 0.00 | 0.50 + (0.00*0.350) = 0.50 |
|  |  | 0 |  |  |  |  |  |
|  |  | 0 |  |  |  |  |  |
|  |  | 0 |  |  |  |  |  |
|  |  | 0 |  |  |  |  |  |
|  |  | 0 |  |  |  |  |  |
|  |  | 0 |  |  |  |  |  |

Type of event: 0= censored, 1= recrudescence, 2= new infection

**Section B: R script for competing risk analysis**

###################################################################################

# Title: R script to compute Kaplan-Meier and Cumulative Incidence Function using

# example #data 1

###################################################################################

#rm(list=ls())

require(cmprsk);require(survival)

# Example data 1

# RC= Recrudescence, RI= New infection, ACPR= cured

dat<-data.frame(time=c(14,14,15,17,22,22,28,28,33,34,44,52,61,63,63,63,63,63,63,63),

outcome=c("RC","RC","RI","RI","RI","RI","RI","RC","RI","RI","RI","RI","RI","RC","ACPR","ACPR","ACPR","ACPR","ACPR","ACPR"))

#----------------------------------------

# create indicator for competing causes

#---------------------------------------

dat$fstatus<-ifelse(dat$outcome %in% c("RC"),1,

ifelse(dat$outcome=="RI",2,0))

#----------------------------------------------

# Estimate cumulative incidence function (CIF)

#----------------------------------------------

xx_cif<-cuminc(dat$time, dat$fstatus,cencode=0)

timepoints(xx_cif, c(seq(0,63,1)))

#-------------------------

# Compute K-M estimates

#-------------------------

dat$censor<-ifelse(dat$outcome=="RC",1,0)

KM.fit<-survfit(Surv(time, censor)~1, data=dat)

summary(KM.fit, time=c(seq(0,63,1)))

**Section C: R script for estimating efficacy accounting for interval censoring**

###################################################################################

# Title: R script to compute survival estimates accounting for interval censoring

###################################################################################

#rm(list=ls())

require(survival);

#--------------------------------------------------------------------------------

# left= date of last negative parasitaemia

# right= date when recurrent parasitaemia is observed

#

# event= censoring indicator: 0= right censored, 1= event occurred at observed

# time, 2= left censored, 3= interval censored

#

# New infections were treated as right censored for generating survival estimates

# for recrudescences

#--------------------------------------------------------------------------------

int.surv<-Surv(dat1$left, dat1$right, dat1$event,type=c("interval"))

int.surv1<-survfit(int.surv~1, data=dat1)

summary(int.surv1, time=c(28)) # display survival estimates on day 28

**Table 3: Survival estimates generated with and without considering interval censoring using data from Uganda [25]**

| Study site | Treatment | Kaplan-Meier estimates  (ignoring interval censoring) | Survival estimates accounting for Interval censoring |
| --- | --- | --- | --- |
| Apac | AQ+SP | 0.927 [0.888-0.968] | 0.921 [0.881-0.963] |
|  | AS+AQ | 0.896 [0.846-0.948] | 0.889 [0.839-0.943] |
|  | CQ+SP | 0.748 [0.677-0.827] | 0.743 [0.671-0.822] |
|  |  |  |  |
| Arua | AQ+SP | 0.860 [0.803-0.920] | 0.853 [0.796-0.915] |
|  | AS+AQ | 0.921 [0.879-0.965] | 0.915 [0.872-0.96] |
|  | CQ+SP | 0.485 [0.390-0.605] | 0.480 [0.384-0.599] |
|  |  |  |  |
| Jinja | AQ+SP | 0.88 [0.830-0.932] | 0.874 [0.824-0.927] |
|  | AS+AQ | 0.958 [0.928-0.989] | 0.953 [0.921-0.985] |
|  | CQ+SP | 0.611 [0.534-0.698] | 0.606 [0.530-0.694] |
| Tororo |  |  |  |
|  | AQ+SP | 0.820 [0.758-0.887] | 0.825 [0.765-0.890] |
|  | AS+AQ | 0.876 [0.813-0.944] | 0.879 [0.818-0.945] |
|  | CQ+SP | 0.624 [0.530-0.736] | 0.631 [0.538-0.740] |

AQ+SP= amodiaquine+ sulfadoxine-pyrimethamine

AS+AQ= artesunate +amodiaquine

CQ+SP= Chloroquine+ sulfadoxine-pyrimethamine

**Section D: R script for pooling Kaplan-Meier across sites**

#############################################################################

# Title: R script to pooling Kaplan-Meier estimates from multicentre studies

#############################################################################

#rm(list=ls())

require(MetaSurv);require(survival)

#--------------------------------------------

# Estimate K-M for each of the study site

#--------------------------------------------

km.fit<-survfit(Surv(time, censor)~site, data=mydata)

# Extract K-M at weekly points for each of the site

# Day1 is coded up as the first day in metasurv package.

x<-summary(km.fit , time=c(1,2,3,7,14,21,28))

y<-cbind(x$strata,x$time, x$n.risk,x$surv, x$std.err)

colnames(y)<-c("Study","time","risk","surv","stderr")

y<-as.data.frame(y)

# Data for metasurv

Study <- y$Study

Times <- y$time

Survival <- y$surv

NbRisk <- y$risk

#------------------------------

# Naive Kaplan-Meier estimates

#------------------------------

naive.km<-survfit(Surv(time, censor)~1, data= mydata)

#---------------------------------------------------------------

# Pooling K-M estimates from different sites using msurv call

#---------------------------------------------------------------

km.pooled<-msurv(Study , Times , NbRisk , Survival , confidence="Greenwood")

# results of random effects meta analysis

RandomEffectSummary<- km.pooled$summary.random

# results of fixed effects meta analysis

FixedEffectSummary<- km.pooled$summary.fixed

#---------------------------------------------------------------------------

# plot the pooled K-M estimates from fixed effects and random effects model

#---------------------------------------------------------------------------

plot(Times, Survival, type="n", col="grey",

xlim=c(0,31), ylim=c(0.85,1),

xlab="Day of follow-up (days)",

ylab="Survival estimates",

las=1, cex.lab=1.5,cex.axis=1.2,axes=FALSE)

axis(1, c(seq(0,28,7)))

axis(2, c(seq(0.85,1,0.02)), las=1); box()

for (i in unique(sort(Study))){

lines(Times[Study==i], Survival[Study==i], type="s", col="grey")

points(max(Times[Study==i]),

Survival[Study==i & Times==max(Times[Study==i])], pch=15)

}

lines(RandomEffectSummary[,1], RandomEffectSummary[,2], type="s",col="red", lwd=3)

points(RandomEffectSummary[,1], RandomEffectSummary[,3], type="s",col="red", lty=3, lwd=3)

points(RandomEffectSummary[,1], RandomEffectSummary[,4], type="s",col="red", lty=3, lwd=3)

lines(FixedEffectSummary[,1], FixedEffectSummary[,2], type="s",col="green", lwd=3)

points(FixedEffectSummary[,1], FixedEffectSummary[,3], type="s",col="green", lty=3, lwd=3)

points(FixedEffectSummary[,1], FixedEffectSummary[,4], type="s",col="green", lty=3, lwd=3)

# Add naive K-M estimates

lines(naive.km, col="blue", ylab="Survival Estimates", las=1, xlab="Day of follow-up", yalb="Survival estimates")

#legend

legend("bottomleft", c("Naive K-M estimates", "Fixed effects meta analysis", "Random effects meta analysis"), col=c("blue","green","red"), bty="n",lty=1, lwd=2)

**Table 4: Kaplan-Meier estimate of recrudescence artesunate+amodiaquine (AS+AQ) arm [25]**

|  | Kaplan-Meier estimates by study sites ^a^ | | | |
| --- | --- | --- | --- | --- |
| Day | Apac | Arua | Jinja | Tororo |
| 7 | 1.000 | 1.000 | 0.995[0.984-1.000] | 1.000 |
| 14 | 1.000 | 0.988 [0.972-1.000] | 0.989 [0.974-1.000] | 1.000 |
| 21 | 0.944 [0.910-0.980] | 0.921 [0.879-0.965] | 0.977 [0.956-1.000] | 0.950 [0.918-0.985] |
| 28 | 0.896 [0.846-0.948] | 0.921 [0.879-0.965] | 0.958 [0.928-0.989] | 0.876 [0.813-0.943] |

^a^ Kaplan-Meier estimates were estimated assuming the data came from one single study. Patients with new infections, indeterminate outcomes and lost to follow-up were censored when deriving the K-M estimates for recrudescence failures. The pooled estimates are presented in main text (Table 2).

**Section E: Chi-squared test for comparing Kaplan-Meier at fixed time points**

Let $\hat{S}1(t)$ and $\hat{S}2 (t)$ be the survival estimates from two treatment groups at specific time-point *t*. The corresponding $X^{2}$ test statistic for the comparing the difference is given by:

$$X^{2}=\frac{\{{\delta\}}^{2}}{Var(\delta)}$$

where $\delta$ is a difference between these two estimates on complementary log-log scale:

$$\delta=\{ln \left( -\ln\left( \hat{S}1(t) \right) \right)-\ln\left( -\ln\left( \hat{S}2(t) \right) \right)$$

and $Var\left( \delta\right)$ is a variance of this difference. This $X^{2}$ statistic has an approximate chi-squared distribution with 1 degree of freedom.

$Var\left( \delta\right)$ is can be calculated (see below for derivation) as

$$Var\left( \delta\right)=\left\{ \frac{1}{\ln\left( \hat{S}1(t) \right)} \right\}^{2}\frac{1}{{\hat{S}1(t)}^{2}}. Var\left( \hat{S}1(t) \right)+ \left\{ \frac{1}{\ln\left( \hat{S}2(t) \right)} \right\}^{2}\frac{1}{{\hat{S}2(t)}^{2}}. Var(\hat{S}2(t))$$

and the $X^{2}$ statistics is equal to:

$$X^{2}=\frac{{\{ln \left( -\ln\left( \hat{S}_{1}\left( t \right) \right) \right)-\ln\left( -\ln\left( \hat{S}_{2}\left( t \right) \right) \right)\}}^{2}}{\left\{ \frac{1}{\ln\left( \hat{S}1(t) \right)} \right\}^{2}\frac{1}{{\hat{S}1(t)}^{2}}. Var\left( \hat{S}1(t) \right)+ \left\{ \frac{1}{\log\left( \hat{S}2(t) \right)} \right\}^{2}\frac{1}{{\hat{S}2(t)}^{2}}. Var(\hat{S}2(t))}$$

**Expression for computing variance of** $\boldsymbol{\delta}$

The Taylor’s series approximations to the variance of a function g(X) of a random variable is given by the following expression: [Equation 2.8, Collet, p 26 ^[[1]](#footnote-1)^]

$Var\left\{ g\left( X \right) \right\}\approx\left\{ \frac{d g\left( X \right)}{d X} \right\}^{2}Var(X)$ _(1)_

Using (1), we obtain the variance of $\log\left( -\log\left( S(t) \right) \right)$ is equal to

$Var\left\{ \ln\left( -\ln\left( S(t) \right) \right) \right\}\approx\left\{ \frac{1}{\ln\left( S(t) \right)} \right\}^{2}Var(\ln\left( S(t) \right))$ _(2)_

Using the following properties of variance for two independent random variables

$$Var \left( X+Y \right)=Var\left( X \right)+Var\left( Y \right)$$

$$Var\left( aX+b \right)=a^{2}. Var(X)$$

We get that,

$$Var\left( \delta\right)=Var\left[ \ln\left( -\ln\left( \hat{S}1(t) \right) \right)-\log\left( -\ln\left( \hat{S}2(t) \right) \right) \right]$$

$$=Var\left[ \ln\left( -\ln\left( \hat{S}1(t) \right) \right)]+Var [\ln\left( -\ln\left( \hat{S}2(t) \right) \right) \right]$$

From equation (2), we obtain:

$Var\left( \delta\right)=\left\{ \frac{1}{\ln\left( \hat{S}1(t) \right)} \right\}^{2}\frac{1}{{\hat{S}1(t)}^{2}}. Var\left( \hat{S}1(t) \right)+ \left\{ \frac{1}{\ln\left( \hat{S}2(t) \right)} \right\}^{2}\frac{1}{{\hat{S}2(t)}^{2}}. Var(\hat{S}2(t))$ _(3)_

**Stratified test**

Let,

$s=1,2,3,\ldots, m$ be the stratum

$\hat{S}_{1s}\left( t \right)$ be survival estimates for group 1 in stratum *s* at time *t*

A stratified version of the test can be used as outlined in equation 17 of Klein et al (2007) [43].

$$X_{Strat}^{2}=\frac{\left( \sum_{s=1}^{m} \{ln \left( -\ln\left( \hat{S}_{1s}\left( t \right) \right) \right)-\ln\left( -\ln\left( \hat{S}_{2s}\left( t \right) \right) \right)\} \right)^{2}}{\sum_{s=1}^{m} \left\{ \frac{1}{\ln\left( \hat{S}_{1s}(t) \right)} \right\}^{2}\frac{1}{{\hat{S}_{12}}^{2}}. Var\left( \hat{S}_{1s}(t) \right)+ \left\{ \frac{1}{\log\left( \hat{S}_{2s}(t) \right)} \right\}^{2}\frac{1}{{\hat{S}_{2s}(t)}^{2}}. Var\left( \hat{S}_{2s}(t) \right))}$$

Example data 2

| Drug A | | Drug B | |
| --- | --- | --- | --- |
| time | censor | time | censor |
| 3 | 1 | 14 | 0 |
| 3 | 1 | 14 | 0 |
| 3 | 1 | 21 | 0 |
| 3 | 1 | 21 | 0 |
| 3 | 0 | 21 | 0 |
| 7 | 1 | 21 | 0 |
| 7 | 1 | 21 | 0 |
| 14 | 0 | 21 | 0 |
| 14 | 0 | 21 | 0 |
| 14 | 0 | 21 | 0 |
| 14 | 1 | 21 | 1 |
| 14 | 1 | 28 | 1 |
| 14 | 0 | 28 | 0 |
| 14 | 1 | 28 | 1 |
| 14 | 1 | 28 | 0 |
| 21 | 1 | 28 | 0 |
| 21 | 1 | 28 | 0 |
| 21 | 1 | 28 | 1 |
| 21 | 1 | 28 | 1 |
| 21 | 1 | 28 | 1 |
| 21 | 1 | 28 | 0 |
| 21 | 0 | 35 | 0 |
| 28 | 1 | 35 | 0 |
| 28 | 0 | 35 | 0 |
| 28 | 0 | 35 | 0 |
| 35 | 0 | 35 | 0 |
| 35 | 0 | 35 | 0 |
| 35 | 0 | 35 | 0 |
| 35 | 0 | 35 | 1 |
| 35 | 0 | 35 | 1 |
| 42 | 0 | 35 | 1 |
| 42 | 0 | 35 | 1 |
| 42 | 0 | 42 | 0 |
| 42 | 0 | 42 | 0 |
| 42 | 0 | 42 | 0 |
| 42 | 0 | 42 | 0 |
| 42 | 0 | 42 | 0 |
| 42 | 0 | 42 | 0 |
| 42 | 1 | 42 | 0 |
| 42 | 0 | 42 | 1 |

0=censored observation; 1= recrudescence failures

The results of the two approaches to compare the survival curves based on this example are presented in main text (Figure 6 of main text).

**Analysis of Uganda data**

This is illustrated using data on amodiaquine+ sulfadoxine-pyrimethamine (AQ+SP) and artesunate +amodiaquine (AS+AQ) arms from four sites using data from Uganda [25]. First the two drugs are compared within each site at a fixed time point of day2 8 using the approach of Klien et al (2007) [41] as described above (see Table 3 on main text). The Kaplan-Meier and its standard error on day 28 can be used to estimate $\hat{\sigma}^{2}$ as presented in table below:

**Table 5:**

| Site | Drug | **A**  Day 28 K-M | **B**  Std. error (K-M) | **C**  Variance of K-M ${(B}^{2})$ | **D**  $\hat{\sigma}^{2}=\frac{C}{A^{2}}$ |
| --- | --- | --- | --- | --- | --- |
| Tororo | AQ+SP | 0.820 | 0.0328 | 0.001073 | 0.00159513 |
| Tororo | AS3+AQ | 0.876 | 0.0332 | 0.001100 | 0.00143292 |
| Arua | AQ+SP | 0.860 | 0.0298 | 0.000888 | 0.00120154 |
| Arua | AS3+AQ | 0.921 | 0.0219 | 0.000480 | 0.00056529 |
| Jinja | AQ+SP | 0.880 | 0.0260 | 0.000676 | 0.00087293 |
| Jinja | AS3+AQ | 0.958 | 0.0155 | 0.000240 | 0.00026161 |
| Apac | AQ+SP | 0.927 | 0.0204 | 0.000416 | 0.00048449 |
| Apac | AS3+AQ | 0.896 | 0.0258 | 0.000666 | 0.00082987 |

###########################################################################

# Title: R script for comparing Kaplan-Meier on day28 after complementary # log-log transformation using data from Tororo, Uganda [25]

###########################################################################

#rm(list=ls())

require(survival)

#---------------------------

# K-M for group1 and group2

#---------------------------

km1<-0.820; km2<-0.876

#-------------------------------------

# Std. Error corresponding to the K-M

#-------------------------------------

sd1<-0.0328; sd2<-0.0332

# Variance of K-M

v1<-sd1^2; v2<-sd2^2

sigma1_sq<-v1/((km1)^2)

sigma2_sq<-v2/((km2)^2)

#-------------------------

# clog-log transformation

#--------------------------

lkm1<-log(km1);lkm2<-log(km2)

cllkm1<-log(-lkm1);cllkm2<-log(-lkm2)

#----------------------------------------------

# constructing a chi-squared test

#----------------------------------------------

chi.statistic.top<- (cllkm1-cllkm2)^2

chi.statistic.bottom.a<- (sigma1_sq)/((lkm1)^2)

chi.statistic.bottom.b<- (sigma2_sq)/((lkm2)^2)

chi.statistic.bottom<-chi.statistic.bottom.a+ chi.statistic.bottom.b

chi.statistic<-(chi.statistic.top)/(chi.statistic.bottom)

#-----------------------------------------------------------------------

# compute p-value comparing against chi-squared in 1 degree of freedom

#-----------------------------------------------------------------------

(1-pchisq(chi.statistic,1))

**Section F: 95% confidence interval for difference in K-M estimates for non-inferiority studies**

Suppose 50 patients were enrolled in each arm in a randomised control study to demonstrate that treatment B is non-inferior to treatment A with a 10% non-inferiority margin taken *a priori*. There were 8 patients in group A who were censored (*) or experienced the event of interest before day 63 (remaining 42 patients were censored at the end of study follow up on day 63). In group B, there were 12 who were censored or experienced the event of interest before day 63 (with remaining 38 censored on day 63) (shown below).

Group A: 14* 21 25 28* 28* 28* 35 42*

Group B: 14 14* 21* 21 21 28 28 28* 35* 35* 42* 63

On day 63, the K-M estimates are 0.937 for group A and 0.874 for group B. The absolute difference in K-M is 0.063. The effective sample size is calculated by dividing the K-M estimate by the number of patients who reached the study without failures or deviations. This is 42/0.937=44.82 = 45 (rounded to integer) for group A, and 38/0.874=43.45= 44 for group B.

The corrected number of failures for group A will then be: (1-KM) $\times$ effective sample size=0.063 $\times$ 45=2.835 =3. For group B, the corresponding estimate is: 0.126 $\times$ 44 = 5.54= 6.

The 95% confidence interval for the difference is then estimated using Wilson’s method using the corrected number of failures and corrected sample size. This can computed using an online calculator, see <http://vassarstats.net/prop2_ind.html>

The difference and associated 95% CI is: 0.069 [95% CI: -0.0635 to 0.2075]. The 95% CI encompasses the 10% margin and we conclude that treatment B is non-inferior to treatment A.

**Section G: Upper limit of non-inferiority margin for hazards ratio**

Non-inferiority is demonstrated if the 95% CI for the estimated hazards ratio remains below the non-inferiority limit on the relative risk scale computed entirely based on the survival estimates in the reference arm(see Figure 7 of main text and Table 6 below).

**Table 6: Upper limit of non-inferiority margin for relative risk (hazards ratio) for different delta margin in survival scale**

| Efficacy in standard arm | $\Delta=0.01$ | $\Delta=0.02$ | $\Delta=0.03$ | $\Delta=0.04$ | $\Delta=0.05$ | $\Delta=0.10$ |
| --- | --- | --- | --- | --- | --- | --- |
| 0.90 | 1.11 | 1.21 | 1.32 | 1.43 | 1.54 | 2.12 |
| 0.91 | 1.12 | 1.24 | 1.36 | 1.48 | 1.60 | 2.23 |
| 0.92 | 1.13 | 1.26 | 1.40 | 1.53 | 1.67 | 2.38 |
| 0.93 | 1.15 | 1.30 | 1.45 | 1.61 | 1.76 | 2.57 |
| 0.94 | 1.17 | 1.35 | 1.52 | 1.70 | 1.88 | 2.82 |
| 0.95 | 1.21 | 1.41 | 1.63 | 1.84 | 2.05 | 3.17 |
| 0.96 | 1.26 | 1.52 | 1.78 | 2.04 | 2.31 | 3.69 |
| 0.97 | 1.34 | 1.68 | 2.03 | 2.38 | 2.74 | 4.57 |
| 0.98 | 1.51 | 2.02 | 2.54 | 3.06 | 3.59 | 6.33 |
| 0.99 | 2.01 | 3.03 | 4.06 | 5.10 | 6.16 | 11.60 |

$\Delta$= Non-inferiority margin on Kaplan-Meier scale

**Section H: 95% confidence interval for difference in K-M estimates based on complementary log-log transformation**

Let $\delta$ be a difference between two K-M estimates ($\hat{S}1(t), \hat{S}2(t))$at time $t$ on complementary log-log scale:$\delta=\ln\left( -\ln\left( \hat{S}1(t) \right) \right)-\ln\left( -\ln\left( \hat{S}2(t) \right) \right)$. Then 95% confidence interval for$\delta$ is obtained as$\delta\pm1.96\sqrt{\gamma}$, where $\gamma$ is the variance of $\delta$ and can be obtained using formula from Appendix E:

$$\gamma=Var\left( \delta\right)=\left\{ \frac{1}{\ln\left( \hat{S}1(t) \right)} \right\}^{2}\frac{1}{{\hat{S}1(t)}^{2}}. Var\left( \hat{S}1(t) \right)+ \left\{ \frac{1}{\ln\left( \hat{S}2(t) \right)} \right\}^{2}\frac{1}{{\hat{S}2(t)}^{2}}. Var(\hat{S}2(t))$$

Parameter $\delta$ can also be expressed as a logarithm of ratio of cumulative hazards in the two groups:

$$\delta=\ln\left( -\ln\left( \hat{S}1(t) \right) \right)-\ln\left( -\ln\left( \hat{S}2(t) \right) \right)$$

$$\Rightarrow\delta=\ln\left( \frac{-\ln\left( \hat{S}1(t) \right)}{-\ln\left( \hat{S}2(t) \right)} \right)= ln \left( \frac{\hat{H}_{1}(t)}{\hat{H}_{2}(t)} \right)$$

Where $\hat{Hi}\left( t \right)=-ln(\hat{Si}\left( t \right))$ is the cumulative hazard function in group *I* at time t.

The corresponding 95% confidence for the hazards ratio can be constructed as:

$${\{\hat{H}}_{1}\left( t \right)/\hat{H_{2}}(t)\} \exp[ \pm1.96* se (\delta) ]$$

**Example:** The demonstration on non-inferiority based on complementary log log transformation of Kaplan-Meier estimates is illustrated using data presented in Appendix F. The K-M estimates and associated standard error on day 63 is:

| Drug | Day 63 K-M | Standard error of K-M |
| --- | --- | --- |
| Drug A  (reference drug) | 0.937 | 0.035 |
| Drug B | 0.874 | 0.048 |

$$\delta=\ln\left( -\ln\left( \hat{S}1(t) \right) \right)-\ln\left( -\ln\left( \hat{S}2(t) \right) \right)$$

$$=\ln\left( -\ln\left( 0.937 \right) \right)-\ln\left( -\ln\left( 0.874) \right) \right)$$

$$=-0.7239$$

The variance is computed using:

$$Var\left( \delta\right)=\left\{ \frac{1}{\ln\left( \hat{S}1(t) \right)} \right\}^{2}\frac{1}{{\hat{S}1(t)}^{2}} Var\left( \hat{S}1(t) \right)+ \left\{ \frac{1}{\ln\left( \hat{S}2(t) \right)} \right\}^{2}\frac{1}{{\hat{S}2(t)}^{2}} Var(\hat{S}2(t))$$

$${=\left\{ \frac{1}{\ln\left( 0.937 \right)} \right\}}^{2}\times\frac{1}{{0.937}^{2}}\times{0.035}^{2}+ \left\{ \frac{1}{\ln\left( 0.874 \right)} \right\}^{2}\times\frac{1}{{0.874}^{2}}\times{0.048}^{2}$$

$$=0.497$$

95% CI for $\delta$ is:

$$\delta[95\% CI: \delta\pm1.96\times se\left( \delta\right)]$$

$$=0.724 [-0.7239 \pm1.96\times\sqrt{0.497}]$$

$$=0.724 (-2.106, 0.658)$$

This can be exponetiated to hazards ratio scale giving:

$$0.484 (95\% CI:0.122-1.931)$$

For a 10% non-inferiority margin in K-M scale, the corresponding upper limit of hazards ratio for a reference arm with efficacy of 0.94 is 2.82 (Table 6 above). In this example, the upper limit of the derived 95% confidence interval for the hazard ratio is 1.93, which is below the upper limit of 2.82 thus demonstrating non-inferiority.

1. David Collett. Modelling Survival Data in Medical Research. Third Edition. 2015 [↑](#footnote-ref-1)
